# Efficient Removal of Nickel from Wastewater Using Copper Sulfate–Ammonia Complex Modified Activated Carbon: Adsorption Performance and Mechanism

**DOI:** 10.3390/molecules29102405

**Published:** 2024-05-20

**Authors:** Yifei Wang, Xiaoxiao Yan, Yidi Zhang, Xiaoxin Qin, Xubiao Yu, Li Jiang, Bing Li

**Affiliations:** 1 School of Civil & Environmental Engineering and Geography Science, Ningbo University, Ningbo 315211, China; yifei6456@gmail.com (Y.W.); yanxiaoxiao1@foxmail.com (X.Y.); 15034072885@163.com (Y.Z.); qinxiaoxin0412@163.com (X.Q.); yuxubiao@nbu.edu.cn (X.Y.); 2 Department of Civil and Environmental Engineering E4130 Engineering Gateway Building, University of California, Irvine, CA 92697-2175, USA; 3 College of Environmental and Resource Science, Zhejiang University, 866 Yuhuangtang Rd, Hangzhou 310058, China

**Keywords:** activated carbon, modification, copper sulfate–ammonia complex, nickel removal, adsorption mechanism

## Abstract

The necessity to eliminate nickel (Ni) from wastewater stems from its environmental and health hazards. To enhance the Ni adsorption capacity, this research applied a copper sulfate–ammonia complex (tetraamminecopper (II) sulfate monohydrate, [Cu(NH_3_)_4_]SO_4_·H_2_O) as a modifying agent for a Phragmites australis-based activated carbon preparation. The physiochemical properties of powdered activated carbon (PAC) and a modified form ([Cu(NH_3_)_4_]-PAC) were examined by measuring their surface areas, analyzing their elemental composition, and using Boehm’s titration method. Batch experiments were conducted to investigate the impact of various factors, such as Ni(II) concentration, contact time, pH, and ionic strength, on its substance adsorption capabilities. Additionally, the adsorption mechanisms of Ni(II) onto activated carbon were elucidated via Fourier-transform infrared (FTIR) spectroscopy and X-ray photoelectron spectroscopy (XPS). The findings indicated that modified activated carbon ([Cu(NH_3_)_4_]-PAC) exhibited a lower surface area and total volume than the original activated carbon (PAC). The modification of PAC enhanced its surface’s relative oxygen and nitrogen content, indicating the incorporation of functional groups containing these elements. Furthermore, the modified activated carbon, [Cu(NH_3_)_4_]-PAC, exhibited superior adsorption capacity relative to unmodified PAC. Both adsorbents’ adsorption behaviors conformed to the Langmuir model and the pseudo-second-order kinetics model. The Ni(II) removal efficiency of PAC and [Cu(NH_3_)_4_]-PAC diminished progressively with rising ionic strength. Modified activated carbon [Cu(NH_3_)_4_]-PAC demonstrated notable pH buffering and adaptability. The adsorption mechanism for Ni(II) on activated carbon involves surface complexation, cation exchange, and electrostatic interaction. This research presents a cost-efficient preparation technique for preparing activated carbon with enhanced Ni(II) removal capabilities from wastewater and elucidates its underlying adsorption mechanisms.

## 1. Introduction

Recent decades have seen heavy metal contamination in aquatic ecosystems becoming a critical global concern, jeopardizing ecosystem integrity and public health [1]. Due to their non-biodegradable nature, heavy metals tend to accumulate in living organisms, leading to biomagnification through the food chain [2]. Nickel (Ni), a detrimental heavy metal, is present in wastewater from various industries including plastic electroplating, mineral processing, leather tanning, paint and battery production, metal surface treatment, and the enamel sector [3,4]. The World Health Organization (WHO) reports that prolonged exposure to environments with soluble Ni(II) concentrations between 1 and 10 mg/m^3^ correlates with an increased likelihood of developing pulmonary fibrosis, renal diseases, edema, and neurodegenerative disorders in humans [5,6]. Given the environmental and health hazards associated with Ni(II), its removal from wastewater is crucial and imperative.

Ni(II) can be removed from aqueous solutions through physicochemical methods such as co-precipitation, electrochemical methods, froth flotation, ion exchange, membrane separation, and adsorption [1,7,8,9]. Due to its highly efficient, low-cost, and simple operation, adsorption is a prevalent method for Ni removal from wastewater [10,11]. Common adsorbents include carbonaceous waste materials and polymeric and mineral adsorbents [11]. Among adsorbents, activated carbon stands out due to its extensive surface area, porous structure, and surface functional groups [12]. Furthermore, various countries and regions can adapt to local conditions when studying adsorbents and select the most extensive and appropriate local materials to make activated carbon. Iamsaard et al. [13] and Deng et al. [14] developed activated carbon from pineapple leaves and rice straw, respectively, targeting Ni adsorption, and repotted maximum capacities of 44.88 mg/g and 27.31 mg/g, respectively. Advancing the development of activated carbon from waste materials not only promotes waste utilization but also expands options for Ni adsorption.

Modification alters the hydrophilicity/hydrophobicity, surface area, surface charge, and surface functional groups of activated carbon [11]. To further improve adsorption capacity, researchers have developed a number of methods to modify activated carbon with chemical agents, such as acid treatment [15], base treatment [16], salt treatment [5], impregnation [17], and grafting [18]. Nitrogen-doped activated carbon, prepared from nitrogen-rich precursors (e.g., urea, melamine, hexamethylenetetramine [19]) or ammonia gas post-treatment [20], enhances heavy metal ion removal [21,22]. Utilizing sucrose and carbon materials as substrates and NH_4_Cl for nitrogen doping, a porous activated carbon variant was synthesized, exhibiting high removal efficiencies for Zn^2+^ (87%), Cd^2+^ (82%), and Pb^2+^ (95%) [23]. Dinh et al. [24] prepared nitrogen-doped porous biochar by air oxidation, which enhanced the Ni(II) adsorption capacities from 3.85 mg/g (pristine activated carbon) to 8.13 mg/g (nitrogen-doped activated carbon). Activated carbon with high nitrogen content derived from waste leather can remove the Ni from aqueous solution with an adsorption amount of 106.0 mg/g [25]. Therefore, preparing nitrogen-doped activated carbon is a promising method.

The US Environmental Protection Agency (EPA) classifies copper (Cu) as a priority pollutant [26]. Recycling and reusing copper hold significant importance as they can mitigate its environmental impact and enhance sustainability within industries [27]. Copper is frequently employed in the modification of adsorbents [28,29]. For instance, activated carbon modified with 0.25 M CuO demonstrated the greatest adsorption capacity for propanethiol [30], while CuCl_2_ impregnation enhanced the Hg^0^ adsorption capacity of coconut-shell-based activated carbon from 95.8 to 631.1 µg/g [28]. To enhance the adsorption capacity, it is imperative to identify and utilize the most suitable and efficient modifying agent that optimizes the adsorption potential of activated carbon. Consequently, the modification effect of copper sulfate–ammonia complex on activated carbon is worth further study.

In the present work, the copper sulfate–ammonia complex tetraamminecopper(II) sulfate monohydrate ([Cu(NH_3_)_4_]SO_4_·H_2_O) was used as the modification agent. This paper aims to investigate and compare the porous structure, surface chemistry, and adsorption properties of modified PAC ([Cu(NH_3_)_4_]-PAC) with those of the original activated carbon (PAC). Through batch experiments, the impacts of initial Ni(II) concentration, ionic strength, and pH on Ni(II) adsorption to both PAC types were assessed. Both unmodified and modified powdered activated carbon (PAC) concentrated on the analyzed surface area, pore features, the point of zero charge, surface functional groups, the state of surface binding, and elemental distribution were characterized to clarify the mechanisms behind Ni(II) adsorption on these adsorbents.

## 2. Results and Discussion

### 2.1. Physiochemical Properties of Adsorbents

**Figure 1:**
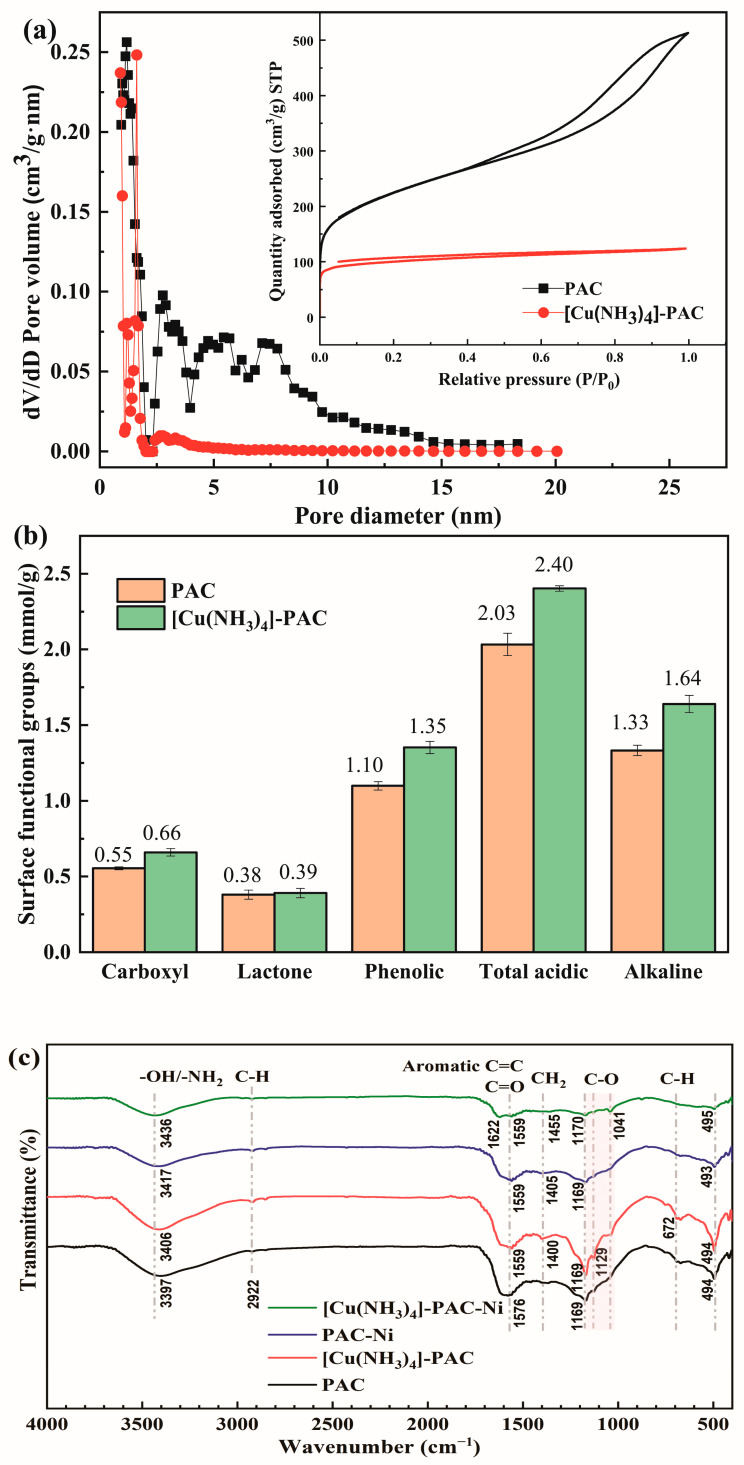
Characterization of PAC and [Cu(NH_3_)_4_]-PAC: (**a**) pore size distribution and nitrogen adsorption–desorption isotherms; (**b**) surface functional group analysis via Boehm’s titration; (**c**) FTIR spectra pre- and post-Ni(II) adsorption.

The adsorption efficiency of Ni(II) is directly influenced by the textural and chemical properties of adsorbents. Both PAC and [Cu(NH_3_)_4_]-PAC exhibit significant pore structure characteristics (Figure 1a). According to the N_2_ adsorption/desorption isotherms, PAC’s classification under the IUPAC falls into the type IV category [31], indicative of a mesoporous structure with pore sizes ranging from approximately 2 nm to 20 nm. However, the structure of [Cu(NH_3_)_4_]-PAC with a pore size of 2–5 nm belonged to the type I isotherm, which was usually used to describe the adsorption on microporous adsorbents [31]. Similarly, the V_mes_/V_TOT_ of PAC and [Cu(NH_3_)_4_]-PAC were 0.76 and 0.58 (Table S1), respectively, which indicated a reduction of hollow structures on the activated carbon’s surface, which were transformed into smaller pore structures. The PAC exhibits a larger specific surface area (S_BET_ = 769.2 m^2^/g) and total pore volume (V_TOT_ = 0.52 cm^3^/g) compared to [Cu(NH_3_)_4_]-PAC, which shows lower values of 379.6 m^2^/g and 0.25 cm^3^/g, respectively. Wang et al. [32] obtained similar results, in that the pore volume and pore size of the biochar decreased by 3.63% and 5.37%, respectively, after modification. The reduction in pore size, specific surface area, and pore volume following modification can be attributed to the interaction of [Cu(NH_3_)_4_]SO_4_ with surface molecules of activated carbon during the modification process, leading to pore blockage [33].

Elemental analysis was conducted to identify changes in elemental composition pre- and post-modification. For PAC, the element composition was found to be 63.08% C, 33.23% O, 2.43% H, and 1.08% N, while for [Cu(NH_3_)_4_]-PAC, the percentages of the same elements were 52.32% C, 43.19% O, 2.24% H, and 2.10% N, respectively. (Table S2). The modification process decreased the C content by 10.76%, which was caused by the increase in the relative oxygen and nitrogen content. The O/C ratio of [Cu(NH_3_)_4_]-PAC (0.83) was much higher than that of PAC (0.53), which indicated that the modification increased the oxygen loading on the surface of [Cu(NH_3_)_4_]-PAC. Oxygen-rich functional groups improve heavy metal adsorption and enhance the hydrophilic nature of activated carbon [24,34]. The [Cu(NH_3_)_4_]SO_4_ used for modification would produce NH_3_ during the pyrolysis process. After modification, the relative nitrogen content increased from 1.08% to 2.10%. The nitrogen functional groups also improved the hydrophilicity and adsorption capacity of activated carbon [24,35].

Considering the crucial role of functional groups in heavy metal adsorption [36], FTIR spectral analysis and a Boehm titration experiment were employed to identify and quantify the functional groups on PAC and [Cu(NH_3_)_4_]-PAC surfaces (Figure 1b,c). The wide peak band observed between 3397 cm^−1^ and 3436 cm^−1^ was assigned to the -NH_2_ or -OH functional groups associated with phenols, alcohols, and carboxylic acids [36,37]. Additionally, the peaks at 672 cm^−1^ and 2922 cm^−1^ were indicative of alkene C-H stretching vibrations and the C-H bonds in lignin, hemicellulose, and cellulose, respectively [37,38]. The peak of aromatic C=C, ketone, or carbonyl C=O appeared near 1559 cm^−1^ [39]. The peaks at 1041 cm^−1^ to 1170 cm^−1^ were indicative of the C-O stretching vibration in ethers [40]. The modification did not cause a change in the types of functional groups. The result of Boehm’s titration is shown in Figure 1b. The total acidic functional groups in [Cu(NH_3_)_4_]-PAC, encompassing carboxyl, lactone, and phenolic groups, amounted to 2.403 mmol/g, exceeding that of PAC (2.033 mmol/g). An elevated concentration of acid groups is advantageous for the removal of heavy metal ions from aqueous solution [37]. Post-modification, the quantity of alkaline groups increased from 1.332 mmol/g to 1.639 mmol/g. Additionally, the variance in acidic and alkaline group concentrations may influence the pH_PZC_ of activated carbon, thereby affecting its adsorption capacity.

### 2.2. Ni(II) Adsorption Isotherm

**Figure 2:**
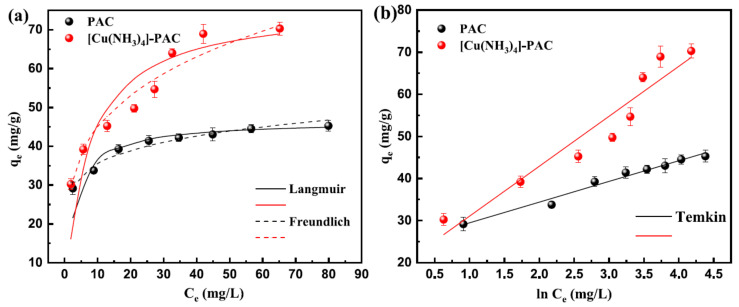
Ni(II) adsorption isotherms for PAC and [Cu(NH_3_)_4_]-PAC: (**a**) Langmuir (solid lines) and Freundlich (dashed lines); (**b**) Temkin model.

The adsorption of Ni(II) onto PAC and [Cu(NH_3_)_4_]-PAC was examined across varying initial concentrations (Figure 2). As the initial Ni(II) concentration (20–100 mg/L) increased, both adsorbents’ capacities initially showed significant growth, followed by a plateau. Lower initial Ni(II) concentrations facilitated reduced transfer resistance between liquid and solid phases, enhancing adsorption. Conversely, higher initial Ni(II) concentrations resulted in increased transfer resistance, yielding a marginal rise in Ni(II) adsorption capacity [41]. Within an initial Ni(II) concentration range of 20 mg/L to 50 mg/L, PAC’s adsorption capacity notably increased from 29.18 mg/g to 41.42 mg/g, and further rose to 45.29 mg/L at an initial concentration of 100 mg/L. For [Cu(NH_3_)_4_]-PAC, when the initial Ni(II) concentration was between 20 mg/L and 70 mg/L, the adsorption capacity significantly grew from 30.27 mg/g to 64.02 mg/g, reaching 70.30 mg/L at a 100 mg/L Ni(II) concentration. This pattern is attributed to the limited availability of vacant sites and active groups on the surface of activated carbon [42]. The initial Ni(II) concentration range corresponding to a remarkable increase in adsorption capacity for [Cu(NH_3_)_4_]-PAC was larger than that of PAC, which indicates that modification increased the adsorption site for Ni(II).

Adsorption isotherm analysis evaluated the adsorption behavior and the connection between initial Ni(II) concentration and capacity [43,44]. The Langmuir, Freundlich, and Temkin models (Supplementary Materials) were used to analyze the data for PAC and [Cu(NH_3_)_4_]-PAC (Figure 2). Calculated parameters were shown in Table 1. According to the correlation coefficients, the adsorption data for both PAC and [Cu(NH_3_)_4_]-PAC align more closely with the Langmuir model than the Freundlich model. Essentially, the Langmuir model suggests adsorption occurs at uniform sites, leading to a single layer of pollutants at saturation [24]. Therefore, Ni(II) adsorption onto PAC and [Cu(NH_3_)_4_]-PAC is characterized as a monolayer chemical adsorption on a uniform surface. The Langmuir constant (K_L_) for PAC and [Cu(NH_3_)_4_]-PAC was 0.347 and 0.143 L/mg, respectively, indicating that [Cu(NH_3_)_4_]-PAC had stronger adsorption capacity than PAC. The Freundlich model suggests adsorption happens on varied surfaces and involves multiple layers [45]. Within this model, the *n* parameter reflects adsorption nonlinearity, with a 1/*n* value from 0.1 to 1 indicating effective Ni(II) adsorption onto PAC and [Cu(NH_3_)_4_]-PAC. The Temkin model views adsorption heat decreasing linearly across the layer as coverage grows, highlighting electrostatic as the main mechanism [46]. The fit of adsorption data to the Temkin isotherm model (R^2^ ≥ 0.91) demonstrates that the energy released during Ni(II) adsorption onto PAC and [Cu(NH_3_)_4_]-PAC diminishes with increased surface coverage.

The maximum Ni(II) adsorption capacity (q_max_) of [Cu(NH_3_)_4_]-PAC, at 76.34 mg/g exceeded that of PAC (46.51 mg/g), suggesting the significance of factors beyond surface area (S_BET_) and total pore volume (V_tot_), such as functional groups, in enhancing adsorption efficiency. The [Cu(NH_3_)_4_]-PAC possessed a stronger q_max_ than other adsorbents, such as bottom ash generated by the combustion of cattle manure (24.6 mg/g) [47], diethylenetriaminepentaacetic acid modification of banana/pomegranate peel (29.240/16.611 mg/g) [48], macroporous poly (vinyl alcohol) (PVA)/chitosan (CS)/Al_2_O_3_ adsorbents (12.03 mg/g) [49], coffee husk-derived biochar composited with MnFe_2_O_4_ nanoparticles (MFO@BC) (5.51 mg/g) [50], modification of biochar with sodium humate acidized by wood vinegar (19.78 mg/g) [41] and activated carbon prepared from Camellia oleifera cake (3.826 mg/g) [16], biogenic magnetite Citrus limetta peel carbon (70.92 mg/g). Meanwhile, some adsorbents were reported to have a better Ni(II) adsorption capacity [51,52], but the precursor of PAC was *Phragmites australis*, harvested from wetland plants, which was beneficial for resource reuse.

### 2.3. Ni(II) Adsorption Kinetics

**Figure 3:**
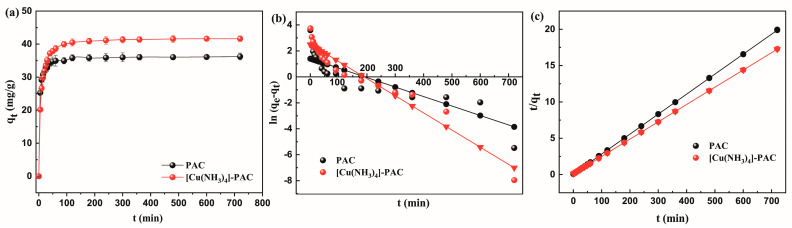
Kinetic analysis of Ni(II) adsorption onto PAC and [Cu(NH_3_)_4_]-PAC: (**a**) adsorption kinetics; (**b**) pseudo-first-order kinetic model fitting; (**c**) pseudo-second-order kinetic model fitting.

Figure 3a illustrates the progression of Ni(II) adsorption capacity at an initial concentration of 30 mg/L, demonstrating an increase in capacity over time until it stabilized at equilibrium. Ni(II) adsorption by PCA achieved equilibrium faster (120 min) compared to [Cu(NH_3_)_4_]-PAC (180 min). The initial 90-min surge in Ni(II) adsorption for both PAC and [Cu(NH_3_)_4_]-PAC results from the swift filling of accessible adsorption sites on the surface of the activated carbon [13]. Between 90 min and 180 min, the rate of Ni(II) adsorption proceeded at a diminished rate due to the decreasing availability of adsorption sites and the diffusion of Ni(II) molecules into the porous structure [37]. Upon reaching equilibrium, [Cu(NH_3_)_4_]-PAC’s adsorption capacity (41.64 mg/g) exceeded PAC’s (36.26 mg/g), demonstrating that modification enhanced Ni(II) adsorption efficiency.

Investigating adsorption kinetics elucidates the efficiency of adsorbents and infers potential adsorption mechanisms [49]. The research employed pseudo-first-order and pseudo-second-order kinetic models to evaluate the rate of adsorption for PAC pre- and post-modification. Table 2 reveals that both PAC and [Cu(NH_3_)_4_]-PAC closely aligned with the pseudo-second-order kinetic model, achieving a prefect correlation coefficient of 1.000, significantly outperforming the results from the pseudo-first-order model (0.666 for PAC, 0.887 for [Cu(NH_3_)_4_]-PAC). The equilibrium adsorption capacity (q_e_) estimated by the pseudo-second-order model closely matched the experimental q_e_ (as shown in Figure 3b,c, suggesting that the adsorption process is primarily driven by chemisorption mechanisms, including surface complexation, ion exchange, and mineral precipitation [53]. Given that k_2_ (g/mg·h) represents the rate constant of the pseudo-second-order adsorption process, the reduction of k_2_ after modification (from 0.011 to 0.005, Table 2 signifies a reduction in the adsorption rate of activated carbon [42]. The adsorption rate of PAC and [Cu(NH_3_)_4_]-PAC was corresponding to the time to reach equilibrium.

### 2.4. Effect of Ionic Strength on Ni(II) Adsorption

**Figure 4:**
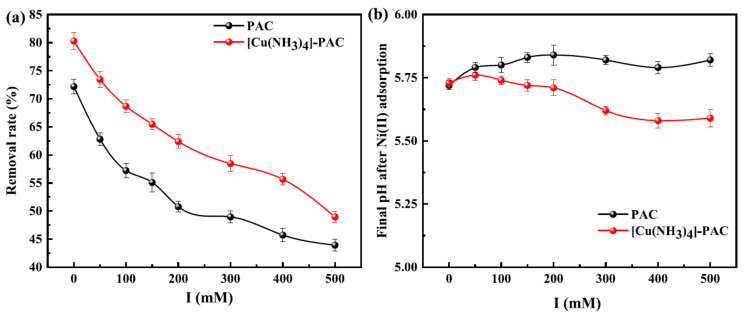
Influence of ionic strength (0, 100, 1000 mM) on (**a**) the removal rate of Ni(II) by PAC and [Cu(NH_3_)_4_]-PAC, and (**b**) the final pH of solution after Ni(II) adsorption.

Figure 4a demonstrates that increasing ionic strength negatively affects the adsorption efficiency of Ni(II) by PAC and [Cu(NH_3_)_4_]-PAC. Specifically, as ionic strength rose from 0 mM to 500 mM, Ni(II) removal efficiency declined from 72.18% to 43.92% for PAC, and from 80.29% to 48.96% for [Cu(NH_3_)_4_]-PAC. This decrease is linked to the increased competition between Na^+^ and Ni(II) ions for adsorption sites at higher ionic strengths, thereby reducing Ni(II) adsorption [32]. This phenomenon highlights the role of ion exchange in the adsorption process of Ni(II) by both PAC and [Cu(NH_3_)_4_]-PAC. Furthermore, the protonation effect induced by NaCl resulted in increased molecular dissociation and enhanced electrostatic attraction during the adsorption process, highlighting complex mechanistic interactions in Ni(II) adsorption under varying ionic strengths [54]. The adsorption of positive charges (Na^+^ or Ni^2+^) onto the surface of activated carbon attenuates the electrostatic attraction towards Ni(II), adversely affecting Ni(II) adsorption. Figure 4b shows that rising NaCl concentrations cause a drop in pH, a result of proton release when Na^+^ and Ni^2+^ interact with acidic functional groups on the activated carbon surface [55]. Furthermore, [Cu(NH_3_)_4_]-PAC, possessing a higher density of acidic functional groups compared to PAC (as shown in Figure 1b), exhibited a more pronounced decrease in pH, highlighting the differential impact of surface chemistry on adsorption dynamics in varying ionic environments.

### 2.5. Effect of pH on Ni(II) Adsorption

**Figure 5:**
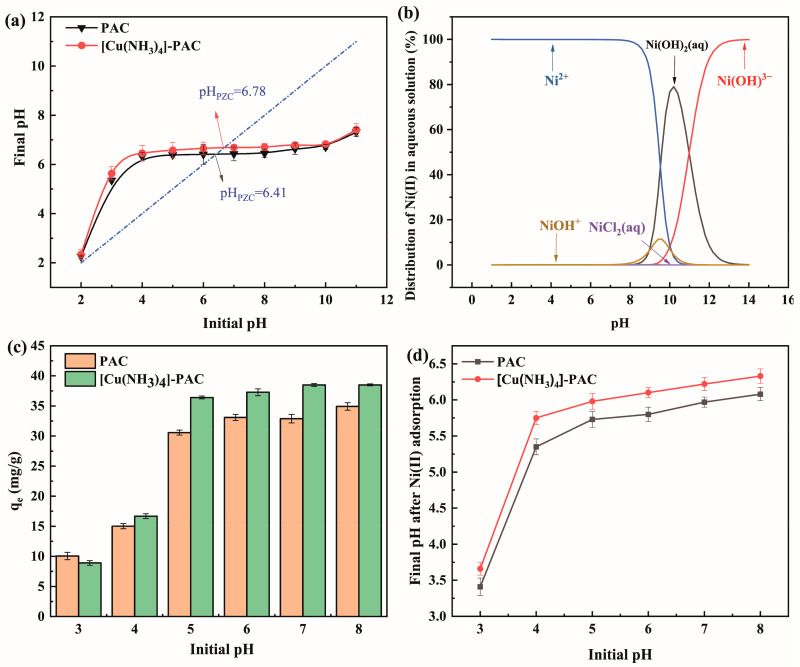
(**a**) Point of zero charge (pH_PZC_) for PAC and [Cu(NH_3_)_4_]-PAC (the dotted lines indicates that initial pH was equal to final pH); (**b**) Ni(II) species distribution as a function of pH at 30 mg/L; (**c**) effect of pH on Ni(II) adsorption by PAC and [Cu(NH_3_)_4_]-PAC; (**d**) pH variations post-Ni(II) adsorption with PAC and [Cu(NH_3_)_4_]-PAC.

The pH level of the solution significantly impacts Ni(II) adsorption by influencing both Ni(II)’s form in the solution and the charge density of the adsorbents’ materials. At the point of zero charge (pH_PZC_), activated carbon exhibit a net charge of zero [56]. When the pH is above pH_PZC_, activated carbon lose protons from their surface, becoming negatively charged and thereby attracting cations. Below pH_PZC_, activated carbon gain protons, adopting a positive charge, which aids in anion adsorption. The pH_PZC_, where the final pH matches the initial pH, is depicted in Figure 5a, with PAC having a pH_PZC_ of 6.41 and [Cu(NH_3_)_4_]-PAC at 6.78. The modification increases pH_PZC_ due to the addition of alkaline groups on the [Cu(NH_3_)_4_]-PAC surface, facilitating more protonation. Figure 5b shows how Ni(II) species vary with pH levels. To maintain, Ni(II) predominates as Ni^2+^ and minimizes the formation of species like Ni(OH)_2_(aq), Ni(OH)_3_^−^, NiCl^+^, NiCl_2_(aq), and NiOH^+^; the studies were performed at pH levels of 8.00 or lower.

The impact of initial pH on adsorption efficiency was analyzed by measuring adsorption capacities across initial pH levels from 3.00 to 8.00, and observing shifts in pH after adsorption (Figure 5c,d). At an initial pH of 3.00, PAC adsorption capacity for Ni (10.05 mg/g) exceeded that of [Cu(NH_3_)_4_]-PAC (8.90 mg/g), a discrepancy attributed to adsorbent protonation. Below the point of zero charge (pH_PZC_), H^+^ ions mainly occupied adsorption sites, leading to electrostatic repulsion against Ni^2+^. Additionally, H^+^ ions were bound to oxygen and nitrogen groups via electrostatic forces or hydrogen bonds [57]. Protonation facilitated the migration of a substantial quantity of H^+^ ions to the activated carbon surface, leading to an elevation in solution pH post-adsorption, as evidenced in Figure 5d. Between initial pH values of 4.00 and 5.00, the adsorption capacity of [Cu(NH_3_)_4_]-PAC for Ni(II) surged from 16.67 mg/g to 36.41 mg/g. This reduction in solution H^+^ concentration diminished the electrostatic repulsion between H^+^ ions and metal cations, thereby enhancing Ni(II) adsorption onto activated carbon. Furthermore, increased Ni(II) adsorption at initial pH values below the pH_PZC_ suggests that ion exchange mechanisms, including cation-π and π-π interactions, contributed to the process [56]. At the initial pH of 7.00, the adsorption capacity for [Cu(NH_3_)_4_]-PAC (38.48 mg/g) substantially surpassed that of PAC (32.88 mg/g). Beyond the pH_PZC_, deprotonation of acidic groups on the activated carbon surface unveiled more active sites, thereby elevating adsorption efficiency [58]. Subsequently, the solution’s pH post-adsorption declined due to activated carbon deprotonation, as depicted in Figure 5d. This observation underscores the superior buffering capacity and robust pH adaptability of modified activated carbon.

### 2.6. Adsorption Mechanisms

Derived from the preceding characterizations and batch experiment outcomes, multiple adsorption mechanisms were identified, encompassing surface complexation, cation exchange, and electrostatic interaction.

#### 2.6.1. Complexation with Oxygen-Containing and Nitrogen-Containing Functional Groups

**Figure 6:**
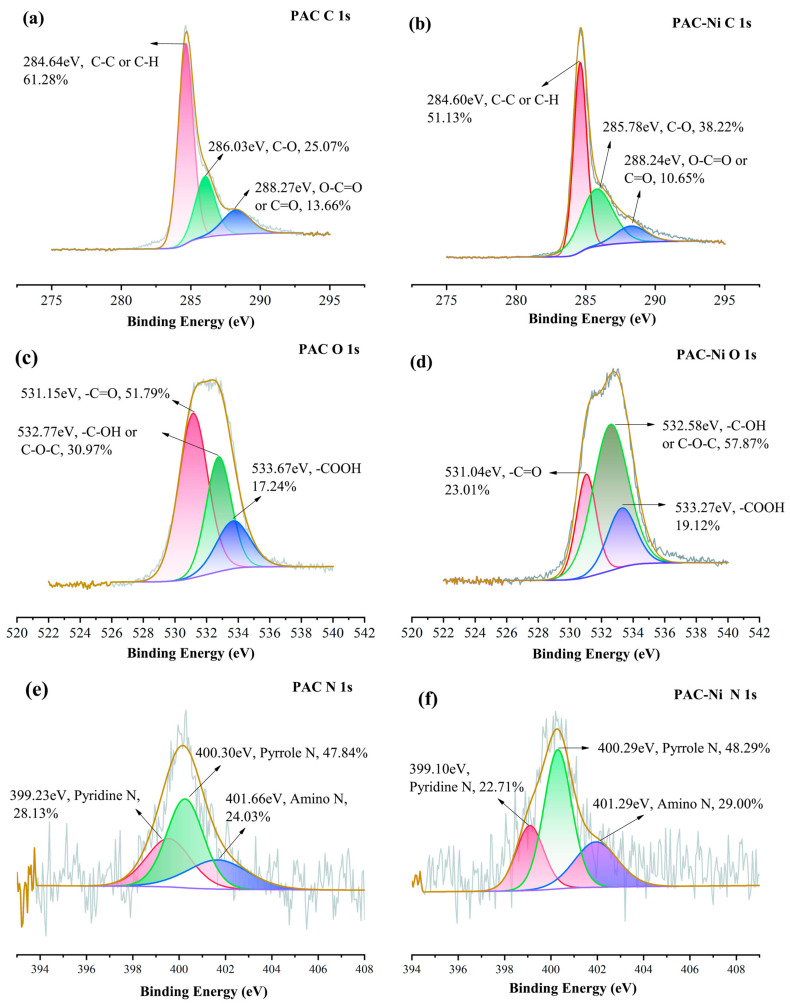
XPS spectra of (**a**) C 1s, (**c**) O 1s, and (**e**) N 1s of PAC before Ni(II) adsorption, and (**b**) C 1s, (**d**) O 1s, and (**f**) N 1s of PAC after Ni(II) adsorption.

**Figure 7:**
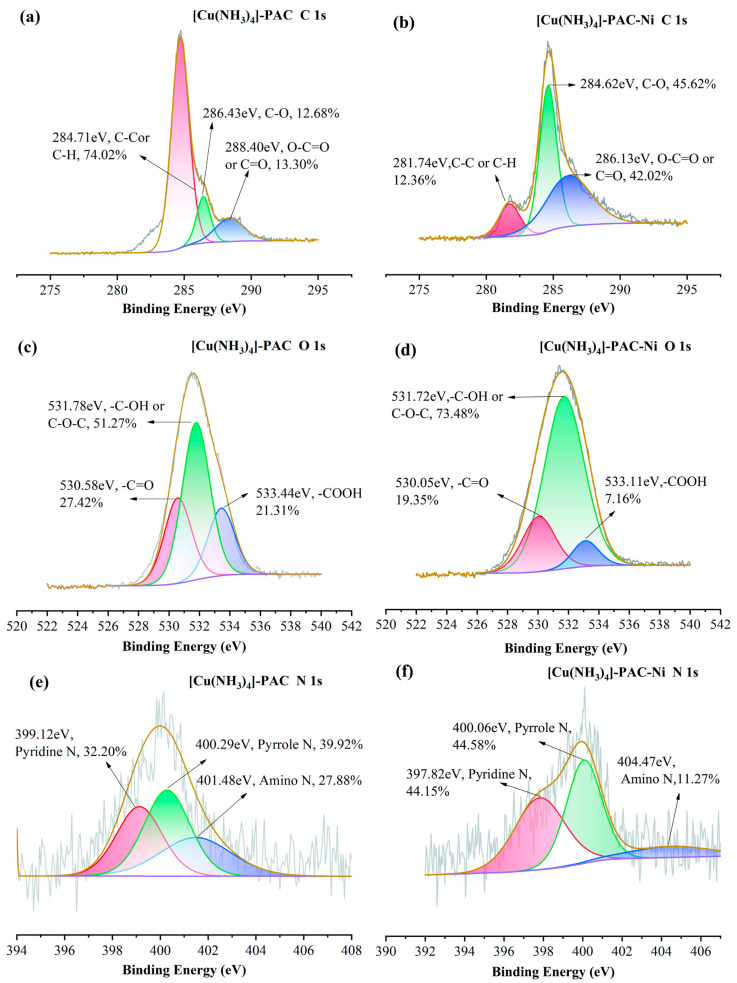
XPS spectra of (**a**) C 1s, (**c**) O 1s and (**e**) N 1s of [Cu(NH_3_)_4_]-PAC before Ni(II) adsorption, and (**b**) C 1s, (**d**) O 1s, and (**f**) N 1s of PAC after Ni(II) adsorption.

**Figure 8:**
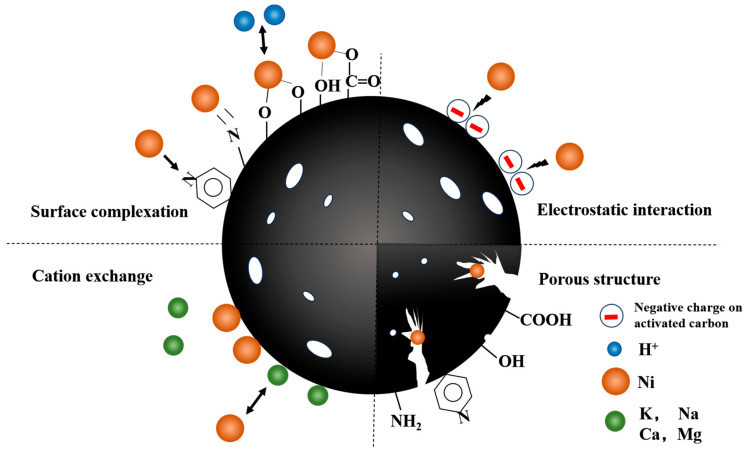
Mechanism of Ni(II) adsorption on [Cu(NH_3_)_4_]-PAC.

To understand the crucial impact of oxygen and nitrogen functional groups on Ni(II) adsorption, we analyzed the pre- and post-adsorption changes in the surface functional groups of PAC and [Cu(NH_3_)_4_]-PAC using FTIR (Figure 1c) and XPS (Figure 6, Figure 7, Figures S1 and S2). XPS survey spectra of PAC and [Cu(NH_3_)_4_]-PAC pre- and post- adsorption (Figure S3) demonstrated a significant presence of C and O. Specifically, for O 1s spectra, the proportion of -C-OH and -COOH functional groups post-modification increased from 30.97% to 51.27% and from 17.24% to 21.31%, respectively, aligning with Boehm’s titration outcomes. Following Ni(II) adsorption, as depicted in Figure 7c,d, the fractions of -C=O and -COOH in [Cu(NH_3_)_4_]-PAC diminished from 27.42% to 19.35% and from 21.31% to 7.16%, respectively. This decrease indicates that these functional groups formed complexes with Ni(II), thereby enhancing the adsorption capacity (Figure 8). Concurrently, the binding energy (BE) of O 1s for [Cu(NH_3_)_4_]-PAC-Ni exhibited a minor reduction, denoting the role of oxygen atoms as electron donors during the adsorption process [37].

The nitrogen-containing functional groups were considered to be another type of functional group that was beneficial for heavy metal adsorption [24]. The nitrogen content in [Cu(NH_3_)_4_]-PAC was quantified at 2.10%, exceeding that of PAC (1.08%), attributable to the ammonia released from [Cu(NH_3_)_4_]-PAC. The N 1s spectra were analyzed and deconvoluted into three individual components: pyridine N (399.1 eV), pyrrole N (400.3 eV), and amino N (401.6 eV) (Figure 6e,f) [24,25]. The modification process enhanced the proportions of pyridine N and amino N from 24.03% to 27.88% and from 28.13% to 32.20%, respectively. Following Ni(II) adsorption, the proportion of pyridine nitrogen in PAC dropped from 28.13% to 22.71%, a decrease linked to chemical bonding between Ni(II) and nitrogen functional groups [10]. For [Cu(NH_3_)_4_]-PAC, amino nitrogen decreased from 27.88% to 11.27% post-Ni(II) adsorption, underscoring the significant role of amino nitrogen in the effective removal of Ni(II) [25].

#### 2.6.2. Cation Exchange

Cation exchange represents a crucial mechanism in the adsorption of heavy metals onto activated carbon. Experiments varying pH and ionic strength demonstrated that post-Ni(II) adsorption pH changes were indicative of Ni(II) ions displacing hydrogen atoms from hydroxyl and carboxyl groups [52]. Additionally, certain cations present on the surface of activated carbon, such as K^+^, Na^+^, Ca^2+^, and Mg^2+^, can be exchanged with Ni(II) ions (Figure 8). Notably, activated carbon derived from Phragmites australis was found to contain significant amounts of K (42.16 g/kg), Na (0.25 g/kg), Ca (3.34 g/kg), and Mg (1.33 g/kg), highlighting the substrate’s potential for cation exchange in heavy metal adsorption processes [34]. Following Ni(II) adsorption, the release of K^+^, Na^+^, Ca^2+^, Mg^2+^, and Cu^2+^ was quantified, revealing that potassium was discharged at the highest rate, with concentrations of 1.873 mg/L for PAC and 1.814 mg/L for [Cu(NH_3_)_4_]-PAC (Table S3). This outcome aligns with the abundant potassium content in Phragmites australis-derived activated carbon. The negligible release of Cu^2+^ (Table S3) suggests a minimal role of Cu^2+^ in the cation exchange process. The XPS spectra (Figure S2) did not exhibit a distinct peak for Cu 2p, implying a low Cu presence on the activated carbon surface. Furthermore, K^+^ and Na^+^ predominantly adhered to the activated carbon surface via electrostatic attraction, whereas Ca^2+^, Mg^2+^, and Cu^2+^ were retained primarily through precipitation or complexation with oxygen-containing functional groups [37].

#### 2.6.3. Electrostatic Interaction

Experiments that varied initial pH levels helped clarify the significance of electrostatic interactions in Ni(II) adsorption, showing the influence of both the solution’s pH and the adsorbent’s point of zero charge (pH_PZC_) on the adsorbent’s surface charge [59]. Electrostatic forces play a key role in enabling Ni(II) ions to attach to the pores of activated carbon during adsorption. Yet, when the pH of the solution is significantly lower than the adsorbent’s pH_PZC_, electrostatic repulsion between Ni(II) ions and the adsorbent occurs, negatively impacting adsorption efficiency [24]. The pronounced disparity in adsorption capacity observed between pH = 3 and pH = 5 underscores the predominance of electrostatic attraction or repulsion as a key mechanism in Ni(II) adsorption (Figure 8) [25]. In addition, the -CH and C=C functional groups present in activated carbon facilitate the adsorption of heavy metals via π-electron coordination [60]. The C 1s XPS spectra revealed an increase in the proportion of C-C and C-H groups on the activated carbon surface from 61.28% to 74.02% following modification (Figure 6a and Figure 7a). Subsequent to Ni(II) adsorption, the share of C-H on the activated carbon surface decreased from 74.02% to 12.36% (Figure 7a,b), suggesting the involvement of Ni(II)-π interactions in the Ni(II) adsorption process.

### 2.7. Environmental Implications

Modification of activated carbon with [Cu(NH_3_)_4_]SO_4_ has enhanced its Ni(II) adsorption capacity, primarily through the incorporation of oxygen- and nitrogen-containing functional groups. The raw materials for activated carbon preparation were *Phragmites australis*, widely utilized in constructed wetland. Phragmites australis, sourced economically and abundantly, was harvested to mitigate biomass decay during winter, facilitating the reuse of this biomass in favor of resource recycling. Furthermore, the modifying agent ([Cu(NH_3_)_4_]SO_4_·H_2_O) can be synthesized from copper sulfate and ammonia solution, with copper sulfate being recoverable from copper-containing wastewater, thereby promoting the circular economy [61]. Nitrates can be transformed into ammonia via electroreduction [62], with ammonia subsequently recoverable in situ using flow coupling devices [63]. The modified activated carbon ([Cu(NH_3_)_4_]-PAC) demonstrates potential for the removal of Ni from Ni-enriched wastewater, including that from electroplating and enamel processing sectors [3]. This research *p* expands the array of adsorptive materials available for Ni remediation.

## 3. Materials and Methods

### 3.1. Reagents and Materials

This research utilized analytical-grade chemicals and distilled water solution preparations. Tetraamminecopper(II) sulfate monohydrate ([Cu(NH_3_)_4_]SO_4_·H_2_O) was procured from Sigma-Aldrich (St. Louis, MO, USA). A 500 mg/L Ni(II) stock solution was prepared by dissolving nickel dichloride (NiCl_2_) in distilled water, and working solutions were subsequently diluted from this stock. The pH value was adjusted using 0.1 M hydrochloric acid (HCl) and 0.1 M sodium hydroxide (NaOH). Sodium chloride (NaCl) was employed to adjust the ionic strengths. *Phragmites australis* was harvested from Baiyun Lake (117.40 °E, 36.86 °N, Shandong Province, China) and washed five times with distilled water. Then, *Phragmites australis* was dried at 105 °C for 48 h to a constant weight and then comminuted into fragments (0.45–1.0 mm).

### 3.2. Adsorbents Preparation

*Phragmites australis* (5 g) was impregnated with phosphoric acid (H_3_PO_4_, 85%) at a 1/2 weight ratio (g Phragmites australis/g H_3_PO_4_) and subjected to a 12-h soak at 25 °C. Subsequently, it was placed in a muffle furnace, heated to 450 °C under limited oxygen for 1 h, and then allowed to cool. The sample was washed with distilled water until a consistent pH was achieved and dried at 105 °C to a constant weight. The resulting activated carbon was sieved to a 100–160 mesh (0.10–0.15 mm) size and labeled as PAC. For the modification of activated carbon, the [Cu(NH_3_)_4_]SO_4_·H_2_O was added to *Phragmites australis* at a ratio of 0.01/1 (g [Cu(NH_3_)_4_]SO_4_·H_2_O/g *Phragmites australis*). Then, the sample was soaked with phosphoric acid (H_3_PO_4_, 85%) at a 1/2 weight ratio (g Phragmites australis/g H_3_PO_4_) and treated as per the above method. The modified activated carbon was labeled as [Cu(NH_3_)_4_]-PAC.

### 3.3. Adsorbent Properties

The pore structure and surface area of both PAC and [Cu(NH_3_)_4_]-PAC were evaluated using N_2_ adsorption/desorption at 77 K with a surface area analyzer (Quantachrome Instruments, Boynton Beach, USA). Elemental composition (C, O, N, H, and others) was analyzed using a Vario EL III Elemental Analyzer (Elementar, Langen, Germany). A Rigaku D/MAX-YA diffractometer (Rigaku Corporation, Tokyo, Japan) assessed crystallinity. Surface chemical functional groups of activated carbon were analyzed using Fourier-380 Fourier transform infrared spectroscopy (FTIR, Bruker Corporation, Billerica, MA, USA) in the range of 400 cm^−1^ to 4000 cm^−1^ with the KBr pellet method. The C, O states of activated carbon and Ni(II) adsorption states were analyzed by PHI 550 ESCA/SAM X-ray photoelectron spectrometer (XPS, Physical Electronics, Eden Prairie, MN, USA) using Mg Kα radiation, with spectra normalized to C 1s at 284.60 ev. Acidic and basic functional groups were quantified using Boehm’s titration method [64], and the point of zero charge (pH_PZC_) was determined through the pH drifting method [65].

### 3.4. Batch Adsorption Experiments

Batch experiments explored how Ni(II) adsorption was influenced by varying the initial concentration (20–100 mg/L), contact time (0–720 min), pH value (2.5–8), and ionic strength (0–500 mM NaCl). A specified quantity of activated carbon was introduced to a 50 mL Ni(II) solution within 150 mL sealed conical flasks, and agitated at 200 rpm and 25 °C in a temperature-controlled shaker. Post 48-h equilibrium adsorption, samples were filtered using a 0.45 μm membrane. The concentration of Ni(II) pre- and post-adsorption were quantified via PQ 9000 inductively coupled plasma optical emission spectrometer (ICP-OES, Analytik Jena AG, Jena, Germany). Adsorption kinetics, along with the impact of initial pH and ionic strength on Ni(II) removal, were operated by combining activated carbon (0.6 g/L) with the 30 mg/L Ni(II) solution, followed by sampling, filtering, and measurement, as previously outlined.

The adsorption capacity of Ni(II) on PAC and [Cu(NH_3_)_4_]-PAC (q_e_ (mg/g)) were calculated by Equation (1):

(1) $$q_{e}=\frac{\left(C_{0}-C_{e}\right)V}{M}$$

where C_0_ and C_e_ represent the initial and equilibrium concentrations of Ni(II) (mg/L), respectively, V denotes the volume of the solution (L), and M refers to the mass of the adsorbent (g).

### 3.5. Statistical Analysis

The speciation of Ni(II) across varying pH levels in aqueous solutions was simulated using Visual MINTEQ (ver. 3.0, https://vminteq.lwr.kth.se/, accessed on 1 December 2021). Batch experiments, including isotherms, kinetics, and the effect of ionic strength and initial pH, were conducted in triplicate, with results reported as mean ± standard deviation. A control experiment was performed under identical conditions.

## 4. Conclusions

This research applied a new modification method for activated carbon preparation. The modifying agent was Tetraamminecopper(II) sulfate monohydrate ([Cu(NH_3_)_4_]SO_4_·H_2_O). Activated carbon effectively removes Ni(II) from water through adsorption. The modification of activated carbon ([Cu(NH_3_)_4_]-PAC) resulted in a decreased specific surface area (S_BET_) and total pore volume (V_TOT_) compared to unmodified (PAC), with the modification leading to an increased presence of oxygen and nitrogen, indicative of O- containing and N-containing functional group incorporation. [Cu(NH_3_)_4_]-PAC demonstrated a superior adsorption capacity relative to PAC. The adsorption processes for both PAC and [Cu(NH_3_)_4_]-PAC were best described by the Langmuir and pseudo-second-order kinetic models. The Ni(II) removal efficiency for both PAC and [Cu(NH_3_)_4_]-PAC was observed to decline as ionic strength increased. Notably, [Cu(NH_3_)_4_]-PAC exhibited robust buffering capabilities and adaptability to pH variations. The adsorption mechanisms for Ni(II) on activated carbon include surface complexation, cation exchange, and electrostatic interactions. This research outlines a cost-effective method for preparing activated carbon for Ni(II) adsorption from wastewater and elucidates the underlying adsorption mechanisms.

## Figures and Tables

**Table 1:** Isotherm adsorption parameters of Ni(II) on PAC and [Cu(NH_3_)_4_]-PAC, fitted using Langmuir, Freundlich, and Temkin isotherm models.

Model	Parameters	Unit	Samples	
			PAC	[Cu(NH_3_)_4_]-PAC
Langmuir	q_max_	mg/g	46.512	76.336
	K_L_	L/mg	0.347	0.143
	R^2^		0.999	0.979
Freundlich	K_F_	mg^(1−1/^*^n)^* L^1/^*^n^*/g	26.073	25.196
	1/*n*		0.133	0.249
	R^2^		0.972	0.964
Temkin	A_T_	L/mg	1.221	1.858
	b	J/mol	99.232	127.145
	R^2^		0.978	0.917

**Table 2:** Kinetic parameters of the pseudo-first order model, pseudo-second order model, and intra-particle diffusion model for the adsorption of Ni(II) by PAC and [Cu(NH_3_)_4_]-PAC.

Kinetic Models	Parameters	Unit	PAC	[Cu(NH_3_)_4_]-PAC
Experimental	q_e, exp_	mg/g	36.256	41.640
Pseudo-first-order	q_e, cal_	mg/g	4.065	12.185
	k_1_	1/min	0.007	0.013
	R^2^		0.666	0.887
Pseudo-second-order	q_e, cal_	mg/g	36.364	42.017
	k_2_	g/(mg·h^)^	0.011	0.005
	R^2^		1.000	1.000

## Data Availability

Data are contained within the article and Supplementary Materials.

## Supplementary Materials

The following supporting information can be downloaded at: https://www.mdpi.com/article/10.3390/molecules29102405/s1, Figure S1: The XPS survey spectra of (a) PAC before and after Ni(II) adsorption, (b) [Cu(NH_3_)_4_]-PAC before and after Ni(II) adsorption; Figure S2: The Cu 2p spectra of [Cu(NH_3_)_4_]-PAC before and after Ni(II) adsorption; Figure S3: The XRD spectra of [Cu(NH_3_)_4_]SO_4_, PAC and [Cu(NH_3_)_4_]-PAC; Table S1: Surface areas and pore volume parameters for PAC and [Cu(NH_3_)_4_]-PAC; Table S2: The element content and elemental ratio of PAC and [Cu(NH_3_)_4_]-PAC; Table S3: The release of K, Ca, Na, Mg, and Cu during the process of Ni(II) adsorption on PAC and PAC and [Cu(NH_3_)_4_]-PAC. References [66,67,68,69] are cited in the Supplementary Materials.
