# Supplementary material for: Efficient Removal of Nickel from Wastewater Using Copper Sulfate–Ammonia Complex Modified Activated Carbon: Adsorption Performance and Mechanism
Source: Molecules. 2024 May 20;29(10):2405. doi: 10.3390/molecules29102405 (PMC11124251; doi:10.3390/molecules29102405)
Supplement: Supplementary file 1 [file molecules-29-02405-s001.zip › molecules-2985149-supplementary.docx]

**Supplementary Materials**

Efficient Removal of Nickel from Wastewater Using Copper Sulfate-ammonia Complex Modified Activated Carbon: Adsorption Performance and Mechanism

Yifei Wang^1,2^, Xiaoxiao Yan^1^, Yidi Zhang^1^, Xiaoxin Qin^1^, Xubiao Yu^1^, Li Jiang^1^*, Bing Li^1^*

*^1^ School of Civil & Environmental Engineering and Geography Science, Ningbo University, Ningbo 315211, Zhejiang, Peoples R China.*

*^2^ University of California, Department of Civil and Environmental Engineering E4130 Engineering Gateway Building, Irvine, CA 92697-2175.*

** Corresponding author at School of Civil & Environmental Engineering and Geography Science, Ningbo University, Ningbo 315211, Zhejiang, Peoples R China.*

*E-mail address:* [*jiangli@nbu.edu.cn (Li*](mailto:jiangli@nbu.edu.cn%20%20(Li) *Jiang); Libing@nbu.edu.cn (Bing Li)*

Number of pages = 9

Number of tables = 3

Number of figures = 3

**Figure S1.** The XPS survey spectra of (a) PAC before and after Ni(II) adsorption, (b) [Cu(NH_3_)_4_]-PAC before and after Ni(II) adsorption.

**Figure S2.** The Cu 2p spectra of [Cu(NH_3_)_4_]-PAC before and after Ni(II) adsorption.

**Figure S3.** The XRD spectra of [Cu(NH_3_)_4_]SO_4_, PAC and [Cu(NH_3_)_4_]-PAC.

**Table S1.** Surface areas and pore volume parameters for PAC and [Cu(NH_3_)_4_]-PAC.

| Identifications | S_BET_  (m^2^/g) | S_mic_  (m^2^/g) | S_ext_  (m^2^/g) | V_tot_  (cm^3^/g) | V_mic_  (cm^3^/g) | V_mes_  (cm^3^/g) | D_p_  (nm) |
| --- | --- | --- | --- | --- | --- | --- | --- |
| PAC | 769.175 | 326.862 | 442.313 | 0.516 | 0.123 | 0.393 | 2.683 |
| [Cu(NH_3_)_4_]-PAC | 379.577 | 306.175 | 73.402 | 0.253 | 0.106 | 0.147 | 2.666 |

S_BET_, BET surface area; S_mic_, microspore surface area; S_ext_, external surface area; V_tot_, total pore volume; V_mic_, microspore volume; V_ext_, external volume; D_p_, the mean pore diameter.

**Table S2.** The element content and elemental ratio of PAC and [Cu(NH_3_)_4_]-PAC.

| **Samples** | **C (%)** | **O (%)** | **H (%)** | **N (%)** | **Other (%)** | **H/C** | **O/C** | **(O+N)/C** |
| --- | --- | --- | --- | --- | --- | --- | --- | --- |
| PAC | 63.08 | 33.23 | 2.43 | 1.08 | 0.19 | 0.04 | 0.53 | 0.54 |
| [Cu(NH_3_)_4_]-PAC | 52.32 | 43.19 | 2.24 | 2.10 | 0.15 | 0.04 | 0.83 | 0.87 |

**Table S3.** The release of K, Ca, Na, Mg and Cu during the process of Ni(II) adsorption on PAC and PAC and [Cu(NH_3_)_4_]-PAC.

| **Sample** | **K(mg/L)** | **Ca (mg/L)** | **Na (mg/L)** | **Mg (mg/L)** | **Cu (mg/L)** |
| --- | --- | --- | --- | --- | --- |
| PAC | 1.873 | 0.062 | 0.019 | 0.021 | 0.004 |
| [Cu(NH_3_)_4_]-PAC | 1.814 | 0.079 | 0.025 | 0.018 | 0.006 |

In this study, adsorption equilibrium data at various ionic strengths (0, 100, 1000 mM NaCl) were analyzed by three adsorption isotherm models, including Langmuir (Eq. (S1)), Freundlich (Eq. (S2)) [66] and Temkin (Eq. (S3)) [67] models.

$\frac{C_{e}}{q_{e}}=\frac{1}{K_{L}q_{m}}+\frac{C_{e}}{q_{m}}$ (S1)

$lnq_{e}=lnK_{F} +\frac{1}{n}lnC_{e}$ (S2)

$q_{e}=\frac{RT}{b}lnA_{T}+\frac{RT}{b}lnC_{e}$ (S3)

where C_e_ is the Cd(II) concentration at equilibrium (mg/L), q_e_, is the Cd(II) adsorption amount at equilibrium (mg/g), q_m_ is the maximum adsorption capacity (mg/g), K_L_ is the Langmuir constant (L/mg); The Freundlich constant K_F_ (mg^(1-1/n)^ L^1/n^/g) and n refers to adsorption capacity and favorable degree of the adsorption process, respectively. *R* is the gas constant (8.314 J/(mol·K)) and *T* (K) is the temperature. The Temkin constants *A_T_* (L/mg) and *b* (J/mol) are related to the binding energy and heat of adsorption, respectively.

Three kinetic models, pseudo-first-order mode (Eq. (S4)) [67], pseudo-second-order model (Eq. (S5)) [68] and intraparticle diffusion model (Eq. (S6)) [69], were applied to fit the experimental data.

$ln\left( q_{e}-q_{t} \right)=lnq_{e}-k_{1}t$ (S4)

$\frac{t}{q_{t}}=\frac{1}{k_{2}q_{e}^{2}}+\frac{t}{q_{e}}$ (S5)

$q_{t}=k_{i}t^{1/2}+C$ (S6)

Where *t* (h) is the adsorption time. The *q*_e_ (mg/g) and *q*_t_ (mg/g) are the adsorbed amount at equilibrium and adsorption time *t* (h), respectively. The *k_1_* (1/h) and *k_2_* (g/(mg·h)) are the rate constant of pseudo-first-order model and pseudo-second-order model, respectively. The *k_i_* (mg/(g·h^1/2^)) and C (mg/g) are the intraparticle diffusion constants, which relate to rate and the thickness of the boundary layer, respectively.
